# Angiosarcoma: a systematic review of biomarkers in diagnosis, prognosis, and therapeutic strategies

**DOI:** 10.3389/fonc.2025.1623327

**Published:** 2025-07-01

**Authors:** Huyen Thuc Tran Luong, Sofie Vercammen, Ario de Marco, Hilde de Rooster, Antonio Cosma

**Affiliations:** ^1^ National Cytometry Platform, Translational Medicine Operations Hub, Luxembourg Institute of Health, Esch-sur-Alzette, Luxembourg; ^2^ Faculty of Science, Technology and Medicine, University of Luxembourg, Esch-sur-Alzette, Luxembourg; ^3^ Small Animal Department, Faculty of Veterinary Medicine, Ghent University, Ghent, Belgium; ^4^ Laboratory for Environmental and Life Sciences, University of Nova Gorica, Nova Gorica, Slovenia

**Keywords:** angiosarcoma, biomarkers, genetic alterations, metabolic pathway, protein, diagnosis, prognosis, therapeutic strategies

## Abstract

Angiosarcoma is a rare, aggressive vascular malignancy characterized by rapid proliferation, early metastasis, and limited therapeutic options, resulting in poor prognosis. The etiopathogenesis of AS remains elusive and diagnosis is challenging due to its similarity to other vascular lesions. This systematic review aims to synthesize existing literature on biomarkers in human AS tissue, encompassing genomic alterations, metabolic pathway changes, specific protein, and their implications for diagnosis, prognosis, and therapy. Eighty-seven studies were identified as meeting predefined eligibility criteria following a systematic search of Pubmed and Embase between 1996 and 2024. The review highlights recurrent mutations (e.g., TP53, POT1, MYC, PTPRB, KDR), altered metabolic pathways (VEGF, ANGPT-TIE, PI3K/Akt/mTOR, MAPK/ERK), and diverse protein expression patterns (e.g., ERG, CD31, CD34, vWF). These biomarkers underscore the complex molecular landscape of AS and offer potential targets for improved diagnostic, prognostic, and therapeutic strategies. This review provides a foundation for further research and the development of novel diagnostic and therapeutic approaches for this challenging malignancy.

**Systematic Review Registration:**

https://www.crd.york.ac.uk/PROSPERO/view/CRD420251019523, identifier (CRD420251019523).

## 1 Introduction

Angiosarcoma (AS) is a rare, aggressive malignant vascular tumor originating from vascular or lymphatic endothelial tissue (1), accounting for up to 2% of all human soft tissue sarcomas (2). It is defined by aggressive proliferation, extensive infiltration of neoplastic cells, and lining abnormal blood-filled spaces (1). The 5-year overall survival rate for advanced-stage AS patients is approximately 30% (3). Patients with metastatic disease exhibit a significantly worse prognosis, with a median survival duration of only 12 months (4). The poor prognosis of AS patients is primarily attributed to early metastases and delayed diagnosis (4). AS prognosis can be influenced by clinical and pathological factors, with high histological grades indicating poor prognosis (5).

The etiopathogenesis of AS remains largely elusive. While the endothelial origin of AS is well-established, there is ongoing debate regarding whether AS originates from blood vessels, lymphatic vessels, or their respective progenitor cells. AS can arise from multiple locations throughout the body due to the ubiquitous presence of endothelial cells (6). The predominant subtype of AS is cutaneous AS, which primarily affects the head and neck area. This is followed by soft tissue AS, which exhibits a highly aggressive clinical behavior (1, 7). AS is subdivided into primary and secondary AS. Primary AS (pAS) can arise in various anatomic sites without a clearly defined etiology. Secondary AS (sAS) is associated with risk factors such as prior radiotherapy, ultraviolet light exposure, chronic lymphedema leading to Stewart-Treves syndrome (8, 9), or exogenous toxin exposure such as vinyl chloride (8, 9), thorotrast (10), arsenic (11), and anabolic steroids (12). Furthermore, various familial genetic syndromes have been linked to AS (13, 14).

Treatment strategies for AS vary depending on the stage and anatomic location of the disease. Localized cutaneous AS is typically managed with wide surgical resection and neoadjuvant/adjuvant radiotherapy, which has shown improved oncological outcomes (15). For advanced or metastatic AS, doxorubicin-based or taxane single-agent chemotherapy regimens are commonly used, although their efficacy outcomes are limited (16). The unfavorable treatment results with conventional therapeutics are exacerbated by late diagnosis and the rarity of AS, which limits the conduct of large-scale randomized controlled trials to establish optimal treatment protocols (17).

The diagnosis of AS is challenging due to its similarity to other vascular lesions, including Kaposi sarcoma, atypical vascular lesions, spindle cell hemangioma, or epithelioid hemangioendothelioma (18). In the absence of distinctive clinical signs, histological findings and immunohistochemical assays have proven to be invaluable tools in the diagnostic process. Thus, the development of specific and sensitive diagnostic biomarkers is critical to improving outcomes for patients with AS. Due to the rarity of AS and the difficulty in conducting extensive cohort studies, we performed a comprehensive systematic review of the existing literature on AS biomarkers, encompassing genomic alteration, metabolic pathway dysregulation, and characteristic protein expression profiles. By classifying these biomarkers, we aim to provide a framework for developing targeted multiplex panels. Such tools would enable simultaneous quantification of relevant proteins, somatic mutations, and pathway activation biomarkers from limited biopsy specimens. The integration of such clinically deployable panels has the potential to personalize diagnostic, prognostic, and therapeutic strategies for this challenging malignancy.

## 2 Methodology

### 2.1 Search strategy

The protocol of this systematic review was registered in PROSPERO (CRD420251019523). We conducted a comprehensive literature search using Pubmed and Embase databases. The search strategy employed the following keywords “biomarkers”, “angiosarcoma”, and “human”. The complete search strategies are detailed in Appendix A. The search covered articles published from 1996 to 2024. This systematic review adhered to the Preferred Reporting Items for Systematic Reviews and Meta-Analysis (PRISMA) guidelines of 2009 (19).

### 2.2 Eligibility criteria

This systematic review included studies that met the following criteria: (1) studies reporting biomarker expression in AS; (2) cohort, case-control, or case-series studies including AS patient samples; (3) studies focused specifically on AS, not vascular tumors in general; (4) studies with a sample size of at least four patients to ensure methodological rigor and relevance; (5) studies published in the English language, and (6) studies with full text available. Two authors (H.T.T.L and S.V) independently assessed the eligibility of studies using Rayyan, a web-based application for screening and selecting studies for systematic review. Disagreements were resolved through consultation with a third reviewer (A.C or H.R). Animal studies, cell line studies, xenograft studies, case reports and case series dealing with less than four patients, reviews, systematic reviews, conference reports, meeting abstracts, protocol paper, letter to journals, and editorials were excluded.

### 2.3 Data extraction and synthesis

From each eligible study, the following data were extracted: sample size, type of samples AS (primary or secondary), anatomic location of AS, biomarker(s), and for each biomarker, positive sample size, pattern of expression, and methods of detection. We compiled the extracted data into a master spreadsheet and subsequently tabulated it based on the data categories presented in this article.

Biomarkers were not only classified into three main sections (genetic alteration, metabolic pathway, protein) but were also grouped into three categories based on their reported associations and potential clinical applications:

- Diagnostic Biomarkers: Molecules or genetic alterations reported to aid in the diagnosis or differential diagnosis of AS, including markers with high sensitivity and specificity for AS compared to other vascular tumors or soft tissue sarcomas.
- Prognostic Biomarkers: Markers significantly associated with clinical outcomes such as overall survival, disease-free survival, or metastasis-free survival in multivariate analyses.
- Therapeutic Biomarkers: Molecules or genetic alterations that predict response to specific treatments, or serve as potential therapeutic targets for AS.

Biomarkers meeting criteria for multiple categories were classified accordingly and discussed in each relevant section.

### 2.4 Quality assessment

To evaluate the methodological quality of the included studies, the Newcastle-Ottawa Scale (NOS) was employed (20). This tool assesses non-randomized studies (cohort and case-control designs) based on three domains: selection of study participants, comparability of groups, and ascertainment of either the exposure or outcome of interest. The NOS assigns a maximum of nine points based on specific criteria within each domain. The overall risk of bias for each study will be categorized as low (7–9 points), moderate (4–6 points), or high (0–3 points). For case-series studies, which lack a comparison group, NOS items related to comparability and adjustment were excluded. Instead, the adapted assessment retained five binary-response items focused on selection, case representativeness, and ascertainment of outcomes and exposure. Studies meeting all five criteria were classified as high quality, those meeting four as moderate quality, and those fulfilling three or fewer as low quality (21).

## 3 Results

### 3.1 Study selection

**Figure 1:**
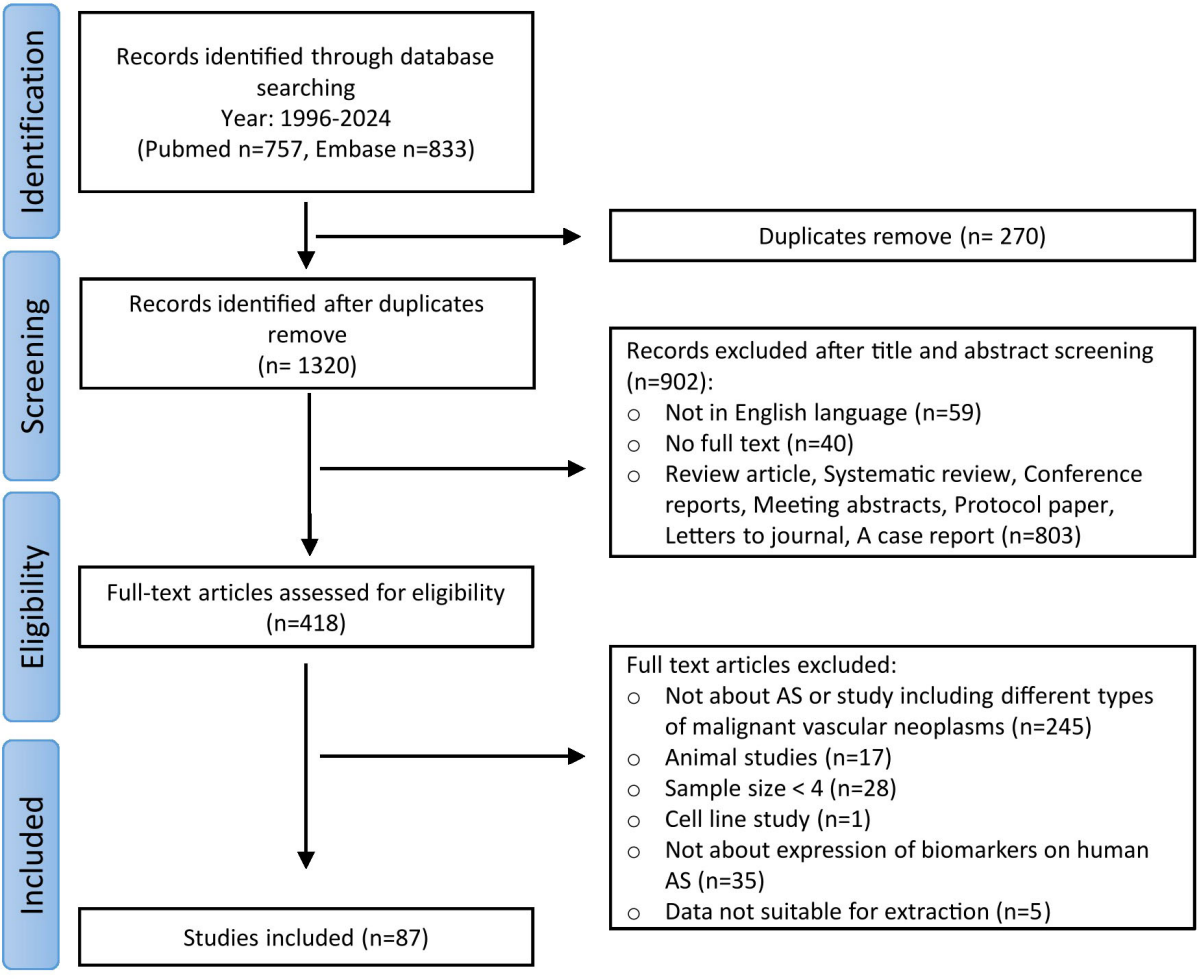
Flowchart of study selection process.

The literature search identified 1590 articles (**Figure 1**). After eliminating duplicated records, 1320 articles were selected for the screening step. Title and abstract screening resulted in the exclusion of 902 articles. Subsequently, 418 articles were assessed for eligibility, with 330 articles being excluded for the following reasons: 245 articles did not specifically address AS or focused broadly on vascular tumors, 17 articles focused on animal studies, 28 had a sample size of less than 4, 1 focused on cell line study, 35 did not study biomarker expression in human AS, and data from 5 articles were unsuitable for extraction. Ultimately, 87 articles were included in this review.

Among the 87 included articles, 14 were case-control studies, 36 were cohort studies, and 37 were case-series studies. Control groups in the case-control studies included healthy individuals, benign or malignant vascular tumors other than AS. Characteristics of all included studies are shown in **Supplementary 1**.

### 3.2 Molecular landscape – genetic alterations

Human AS exhibits a wide range of molecular abnormalities. Several studies have recently performed whole genome, exome, transcriptome, or targeted sequencing to characterize the genomic landscape of this malignancy. These investigations have identified recurrent genetic alterations that are likely key tumorigenesis drivers. While there are some similarities in the top mutated genes between studies (e.g., TP53, PIK3CA, KDR, MYC), significant heterogeneity exists in the alteration frequencies and their association with the tumor’s anatomic location. Manner et al. (22), for the first time, demonstrated that primary and secondary AS represent distinct genetic entities despite their morphological similarities. Furthermore, Espejo-Freire et al. identified different genomic profiles based on the AS primary site (23). In this section, we synthesize and summarize existing data regarding the genetic abnormalities of AS (**Table 1**; **Supplementary 2**), providing insights into its complex molecular characterization.

**Table 1:** Summarized data regarding the genetic alteration biomarkers in AS.

Genetic alteration biomarker(s)	Alteration type	Number of AS samples	Number of mutated AS samples	% Positivity (Mean % + Range)	Method	Classification (Diagnostic/Prognostic/Potential therapeutic)	Reference
TP53	Missense mutation In-frame insertion/deletion Nonsense mutation Deletion Frameshift insertion/deletion	329	89	27% (10–69)	NGS, WTS, WES, WGS Surveyor nuclease assay PCR-based DNA sequencing	–	(23, 26–32)
POT1	Missense mutation Amplification	205	34	17% (16-19)	NGS, WTS, WES, WGS	–	(23, 26, 28)
MYC	Missense mutation Amplification	898	376	42% (11-90)	NGS, WTS, WES, FISH	Diagnostic Prognostic	(22, 23, 26, 27, 29, 43–53)
PTPRB	Missense mutation Nonsense mutation Frameshift insertion/deletion	99	18	18% (11-29)	NGS, WES, WGS	–	(26–28)
KDR	Missense mutation In-frame insertion/deletion Splice site Amplification	405	48	12% (7-73)	NGS, WTS, WES, WGS, FISH	–	(23, 26–28, 30, 48, 65)
FLT4	Missense mutation Amplification	477	42	9% (4-18)	NGS, WTS, WES, WGS, FISH Affymetrix Human Exon 1.0 ST array	Prognostic Potential therapeutic	(23, 26–29, 44, 45, 48, 53)
PIK3CA	Missense mutation Frameshift insertion/deletion	303	40	13% (0-45)	NGS, WTS, WES Custom TaqMan^®^ Assay Design Tool	Prognostic	(23, 26, 29, 30, 78, 81)
RAS-RAF-MEK-ERK pathway mutation	Missense mutation Splice site Amplification	373	52	14% (0-53%)	NGS, WTS, WES, WGS Custom TaqMan^®^ Assay Design Tool	–	(23, 26–29, 78, 81)

Detailed data can be found in **Supplementary 2**.

NGS, Next Generation Sequencing; WTS, Whole-transcriptome sequencing; WES, Whole-exome sequencing; WGS, Whole-genome sequencing; FISH, Fluorescence *in situ* hybridization; TP53, Tumor protein p53; POT1, Protection of Telomeres 1; PTPRB, Protein tyrosine phosphatase receptor B; KDR, Kinase Insert Domain Receptor; FLT4, Fms-related tyrosine kinase 4; PIK3CA, Phosphatidylinositol-4,5-Bisphosphate 3-Kinase Catalytic Subunit Alp.

#### 3.2.1 TP53

Located on chromosome 17p13.1, TP53 is a well-established tumor suppressor gene, and its loss of function - whether through downregulation or mutational inactivation – has a robust correlation with oncogenesis (24). Upon activation, TP53 plays a critical role in several intracellular pathways, such as cell cycle arrest to maintain genomic stability, apoptosis, senescence, and ferroptosis to eliminate irreparably damaged cells. As a result, TP53 is frequently termed “the guardian of genome” as it helps prevent the accumulation of oncogenic mutations that could drive malignant transformation (24, 25).

As in other tumors, TP53 abnormalities are common in AS. Most TP53 mutations are missense mutations, resulting in the production of the full-length p53 mutant protein (23, 26–31). In a next-generation and whole-transcriptome sequencing analysis of 143 AS cases, TP53 was identified as the most frequently mutated gene with a mutation frequency of 29% (23), particularly high in head and neck AS (48.8%). Similarly, the Angiosarcoma Project of Painter et al. reported recurrent TP53 mutations in 30% of cases based on whole-exome sequencing conducted on 47 AS specimens (26). Another comprehensive genomic analysis using a sequencing assay targeting 341 established cancer-related genes revealed TP53 mutations in 35% of AS cases (27). Interestingly, Kiyohara et al. reported an even higher mutation frequency, with 69% of AS tissue samples testing positive for TP53 mutation (32). Other studies have also reported frequent TP53 mutations in human AS (28–31). The frequency of TP53 mutations appears to vary depending on tumor location. Naka et al. observed that TP53 gene mutations were more common in the head, heart, and extremities compared to those located in the trunk (33). The presence of p53 mutant protein has been implicated in promoting angiogenesis. Kieser et al. reported that a mutated form of the TP53 gene induces the expression of vascular endothelial growth factor (VEGF), a potent endothelial cell-specific mitogen and key angiogenic factor (34). These findings underscore the importance of TP53 mutations in AS pathogenesis.

#### 3.2.2 POT1

The POT1 (Protection of Telomeres 1) gene encodes a crucial component of the shelterin complex, which interacts directly with telomeres to regulate chromosomal stability. It plays a key role in preventing atypical telomere elongation and chromosomal fusions (35). Located on chromosome 7q31.33 with a length of 120kb, POT1 mutations, both germline and somatic, along with dysregulated POT1 expression, have been identified in several cancer types. The highest prevalence of POT1 alterations has been observed in cutaneous melanoma, non-small-cell lung carcinoma, squamous cell carcinoma, chronic lymphocytic leukemia, and AS (36). Notably, Shen et al. showed that AS exhibits an 11-fold increased likelihood of carrying POT1 mutation compared to other tumors and often contains multiple POT1 mutations (37).

In AS, recent studies have consistently reported POT1 alterations. Espejo-Freire et al. found POT1 alteration in 16% of all cases, predominately in head and neck AS (41.9%) (23). Similarly, Painter et al. and Chan et al. have reported a similar frequency of POT1 mutation (16-19%) (26, 28). Most of these POT1 mutations are missense mutations, potentially altering protein function.

#### 3.2.3 MYC

Located on chromosome 8q24, MYC is a proto-oncogene encoding for a transcription factor. Its deregulation is a well-recognized oncogenic event implicated in various cancers. MYC influences various signal transduction pathways, including cell proliferation, metabolic processes, cellular differentiation, oncogenic transformation, cell cycle progression, and angiogenesis (38). Its oncogenic activation primarily occurs through two mechanisms: gene amplification, observed in a subset of breast carcinomas (39), or gene rearrangement, characteristic of most Burkitt lymphomas (40, 41). Dysregulated MYC expression following ionizing radiation enhances cell proliferation by promoting an inappropriate transition from the G1 to S phase, resulting in its function as an oncogene (42). Elevated MYC amplification is a characteristic feature of most post-radiation and chronic lymphedema-associated AS (22, 43–47), whereas it is present in only a minor subset of pAS cases (26, 27, 29, 48–53). MYC amplification is also helpful in distinguishing AS from the atypical vascular lesions, which also occur following radiation therapy but have a benign behavior (43–46, 51). A recent study has found that MYC amplification in AS enhances the expression of the miR17–92 cluster (54). This upregulation subsequently leads to the repression of thrombospondin-1, a key endogenous angiogenesis inhibitor. Such suppression promotes the uncontrolled proliferation of malignant endothelial cells.

Subsequent sequencing studies have established MYC amplification as a sensitive and highly specific marker for radiation-induced and chronic lymphedema-associated AS compared to pAS (43–47). Some studies even found that high-level MYC amplification is present in 100% of sAS cases (22, 43, 44, 51, 53), highlighting MYC analysis as a crucial diagnosis tool in distinguishing sAS from other vascular lesions. However, several recent studies have identified MYC amplification in a small proportion of pAS cases, indicating that it is not exclusively associated with sAS (26, 27, 29, 48–53). For instance, Shon et al. found MYC amplification and overexpression in a subset of primary cutaneous AS; however, the clinical significance remains unclear as they were not associated with histopathological features or clinical outcomes (50). The study of Huang et al. corroborated these findings, demonstrating MYC amplification in a small subset of pAS (7%), including those affecting the breast and somatic soft tissue (48). Nonetheless, the strong preference for MYC amplification in sAS compared to pAS suggests a distinct pathogenic mechanism in the context of underlying lymphedema or prior radiation.

MYC amplification has also proven valuable in distinguishing AS from other atypical vascular lesions or sarcoma types. The exclusive presence of MYC amplification in sAS has led to the hypothesis that MYC may play a role in the progression of atypical vascular lesions to AS (43–46, 51). Moreover, Fraga-Guedes et al. and Kuba et al. found that MYC amplification was associated with decreased overall survival (OS) compared to those without MYC amplification (46, 47). This finding implies that MYC amplification may not only serve as a diagnostic marker but also as a prognostic indicator in sAS. Given the high frequency of MYC amplification in sAS and its potential correlation with poor prognosis, targeting MYC represents a promising therapeutic approach that warrants further investigation (55).

#### 3.2.4 PTPRB

Located on chromosome 12q15, protein tyrosine phosphatase receptor B (PTPRB), also referred to as vascular endothelial protein tyrosine phosphatase (VE-PTP), is a transmembrane protein tyrosine phosphatase specifically expressed in endothelial cells. PTPRB functions as a negative regulator of angiogenesis by dephosphorylating TIE2, a key receptor involved in vascular development and homeostasis (56, 57). It inhibits VEGFR2, vascular endothelial cadherin (VE-cadherin), and angiopoietin/TIE2 signaling, thereby modulating angiogenic processes (56, 58). Loss-of-function mutations in PTPRB are believed to enhance angiopoietin/TIE2 signaling and active multiple downstream pathways, including PI3K/Akt/mTOR and MAPK pathway (59). This dysregulation can lead to enhanced angiogenesis and vascular remodeling. Notably, *in vitro* models of angiogenesis have shown that PTPRB inhibition enhances angiogenic activity (60). Beyond its role in angiogenesis, PTPRB has been implicated in promoting metastasis of colorectal carcinoma by inducing epithelial-mesenchymal transition (61).

In AS, PTPRB mutations have been identified in 11-29% of cases (26–28). Mutations in PTPRB are believed to disrupt its function, potentially resulting in dysregulated angiogenesis. Indeed, the majority of PTPRB mutations in AS were truncating, including non-sense mutation and frameshift insertion/deletion (26–28). While PTPRB’s role as a negative regulator of angiogenesis is recognized, it is still uncertain whether angiogenesis driven by PTPRB loss can be effectively targeted through pharmacological VEGF inhibition.

#### 3.2.5 Mutation of VEGFR family

**Figure 2:**
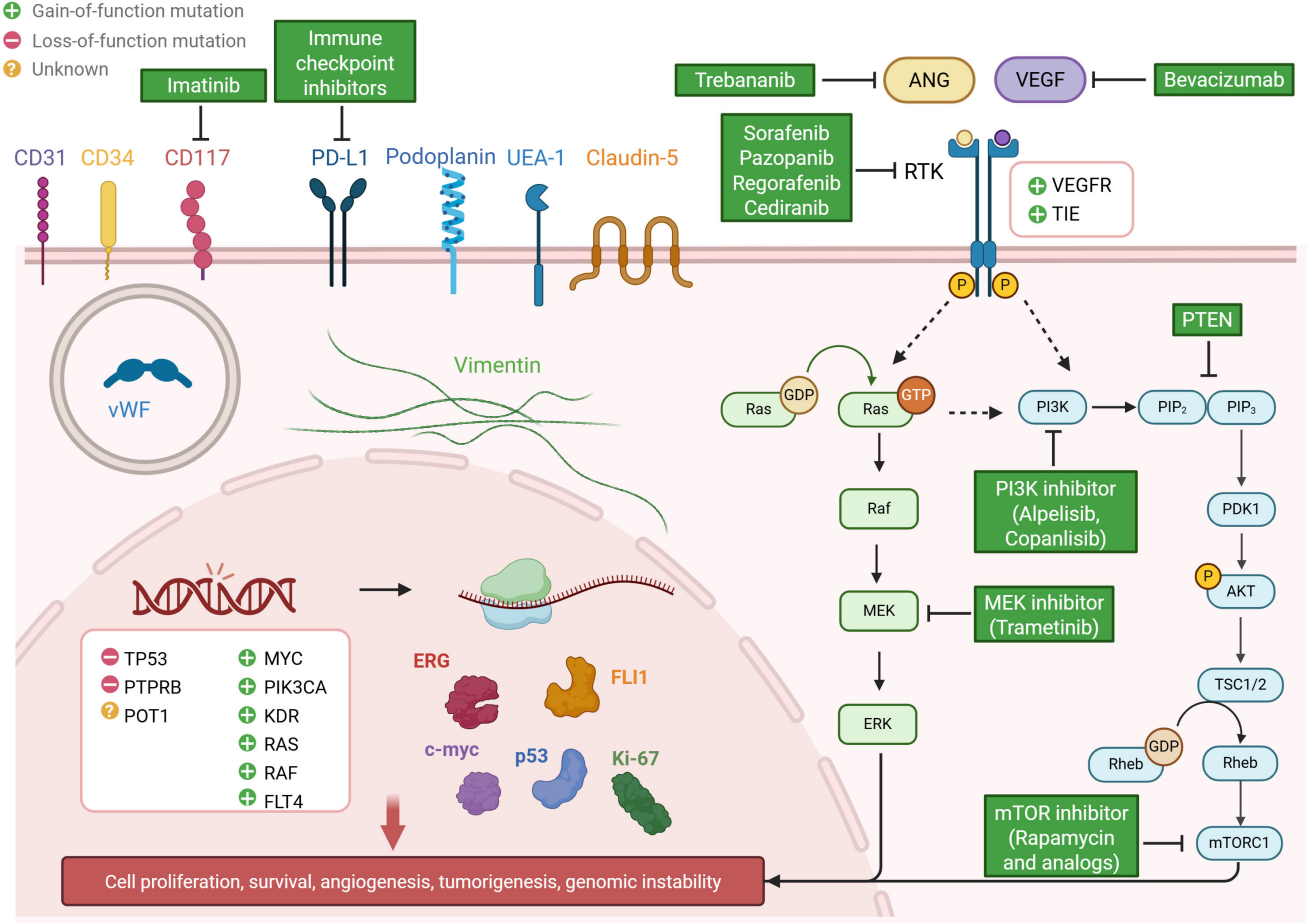
Overview of molecular characterization and potential therapeutic strategies in human angiosarcoma. The figure highlights recurrent genetic alterations (e.g., TP53, POT1, MYC, PTPRB, KDR), metabolic pathway dysregulation (VEGF, ANGPT-TIE, PI3K/Akt/mTOR, MAPK/ERK), and characteristic protein profiles (e.g., ERG, FLI-1, Vimentin, CD31, CD34), alongside corresponding targeted therapies.

The VEGF pathway is a crucial signaling system involved in angiogenesis, vasculogenesis, and vascular permeability. Upon binding of VEGF to its receptors (VEGFRs), it triggers a cascade of intracellular signaling events, including the activation of PI3K/Akt/mTOR and MAPK/ERK pathways, which are essential for promoting cell growth and survival (**Figure 2**). Given the central role of VEGF signaling in vascular development, VEGFRs have been among the most studied potential targets for AS therapy. Notably, genes involved in the VEGF pathway, such as KDR (VEGFR2) and FLT4 (VEGFR3), are frequently amplified and underdo gain-of-function in AS, further underscoring the importance of this pathway in AS pathogenesis.

##### 3.2.5.1 KDR

The KDR gene (Kinase Insert Domain Receptor), also referred to as VEGFR2, is a member of VEGFR family of Receptor Tyrosine Kinase (RTK). Located on chromosome 4q11-12, KDR plays a crucial role in the regulation of both vasculogenesis and angiogenesis through its interaction with various isoforms of VEGF (62). KDR mutations have been implicated in multiple cancers, including colorectal cancer, non-small cell lung carcinoma, breast cancer, and AS (63–65). Given its central role in vasculogenesis, KDR was highly expressed in AS samples at both transcript and protein levels (26, 30, 66, 67).

Recent studies have revealed a high prevalence of KDR gene alterations in primary breast AS. Kuba et al. reported KDR alterations in 73% of primary breast AS cases (30). The Angiosarcoma Project reported KDR mutations at a rate significantly higher than expected by chance, with a 25.5% recurrence rate (26) - notably, 89% of KDR missense mutations observed in primary breast AS samples. KDR mutations were also reported in 10% of AS and were localized specifically to the breast anatomic site, regardless of prior radiation exposure (65). Similarly, Huang et al. found KDR missense mutations in 7% of AS cases, primarily affecting the breast and only present in one case of the lumbar spine (48).

However, other studies have identified KDR mutations in AS at various anatomical locations beyond the breast. For instance, KDR mutations were reported in head and neck, lung, liver, visceral, and extremity AS with incidence rates between 5% and 18% (23, 27, 28). More importantly, the presence of KDR mutations correlated with high protein expression levels, as detected by immunohistochemical analysis (26, 30, 66, 67).

##### 3.2.5.2 FLT4

An additional mechanism of VEGFR activation in sAS involves FLT4 (Fms-related tyrosine kinase 4) amplification. Located on chromosome 5q35, FLT4 encodes the VEGFR3, a RTK activated by VEGF-C and VEGF-D. This receptor is pivotal in regulating the development and maintenance of lymphatic system function (68).

So far, most of the studies have shown that FLT4 amplification primarily occurs in sAS, particularly those associated with radiation therapy for breast cancer or chronic lymphedema (28, 29, 44, 45), and always in association with MYC amplification (27, 29, 44, 45). Guo et al. demonstrated that the gene amplification of FLT4 was found only in sAS with 27% alteration frequency and in association with MYC amplification (44). Similarly, Cornejo et al. reported a similar frequency of FLT4 amplification in sAS, consistently observing co-amplification of FLT4 and MYC across all cases (45). Further investigations also confirmed this co-amplification pattern, suggesting that FLT4 alone may have limited diagnostic value (27, 29, 48). These observations also suggested that FLT4 amplification might be a secondary genetic event following MYC amplification (44). The presence of FLT4 amplification in a subset of radiotherapy-induced AS provides a rationale for exploring tyrosine kinase inhibitors (TKIs) as treatment options. Notably, Guo et al. documented three cases of FLT4-amplified sAS that exhibited either complete or partial response to treatment with sorafenib, which is a multikinase inhibitor (44). Furthermore, FLT4 amplification was correlated with a short OS (48).

However, recent research has challenged the exclusivity of FLT4 amplification to sAS. These studies indicated that FLT4 amplification is not exclusive to sAS, as it was also observed in pAS at various anatomical sites, including the breast and head/neck (23, 26, 48, 53). Interestingly, Espejo-Freire et al. found that in most cases, FLT4 amplification occurred independently of MYC amplification (23).

#### 3.2.6 Mutation of RAS and its downstream signaling pathway

The RAS signaling and its downstream pathway play a pivotal role in the pathogenesis of AS. RAS signaling can be triggered by several cellular receptors, such as RTKs, G-protein coupled receptors (GPCRs), and integrin (69). Activated RAS transduces signals through multiple effector pathways, notably the MAPK cascade and PI3K/Akt/mTOR pathway (70). Dysregulation of RAS signaling is frequently tumorigenic, contributing to uncontrolled endothelial cell proliferation and tumor progression. Among RAS downstream pathways, the dysregulation of the PI3K/Akt/mTOR axis has been frequently documented in AS patients, with evidence suggesting it plays a more important role than the MAPK cascade in disease progression (71, 72).

##### 3.2.6.1 PI3K/Akt/mTOR pathway mutation

The phosphatidylinositol-3-kinase (PI3K)/Akt and the mammalian target of rapamycin (mTOR) signaling pathway constitutes a pivotal intracellular cascade that regulates fundamental cellular processes, encompassing proliferation, growth, survival, and metabolic regulation (73). This pathway exhibits dysregulation in breast cancer and other malignancies, making it a significant target in cancer research (73, 74). PI3K is an oncogene product and functions as a signal transducer that initiates the Akt pathway (75). The PIK3CA gene is responsible for encoding the p110α catalytic subunit of PI3K (76). Among the genetic alterations affecting this pathway, activating mutations in PIK3CA are implicated in promoting uncontrolled cell proliferation and tumor progression. PIK3CA mutations have been reported in various cancer types, including colorectal, breast, liver, brain, stomach, and lung cancer (76, 77). While the prognostic significance of PIK3CA mutations remains debated, most studies support their role in tumorigenesis through hyperactivation of PI3K/Akt/mTOR signaling (76). In AS, PIK3CA mutations are predominantly missense mutations (23, 26, 29, 30). The prevalence of PIK3CA mutations in AS varies across studies, ranging from 6% to 45%. Interestingly, these mutations are almost exclusively found in primary breast AS (26, 29, 30). However, Verbeke et al. found no hotspot PIK3CA mutation in AS of bone or soft tissue, suggesting that PIK3CA mutation for AS may be tumor-location dependent (78). The small-cohort analyses of Kuba et al. indicated that PIK3CA mutations correlated with worse prognosis in AS (30). Given the potential role of PI3K in tumor progression, PI3K inhibition has emerged as a promising therapeutic strategy in PIK3CA-mutant breast AS. Notably, the efficacy of Alpelisib and other PI3K inhibitors warrants further evaluation in primary breast AS to determine their therapeutic potential (79).

##### 3.2.6.2 RAS-RAF-MEK-ERK pathway alteration

The intracellular RAS-RAF-MEK-ERK signaling cascade, known as the MAPK/ERK pathway, is classified as a mitogen-activated protein kinase (MAPK) pathway. Hyperactivation of various elements of this pathway plays a crucial role in several tumors, including AS (80). Emerging evidence suggests that genetic alterations leading to MAPK/ERK pathway dysregulation (e.g., RAS, BRAF) facilitate complex interactions between tumor cells, the tumor microenvironment, and the immune system (69). Persistent MAPK activation enables uncontrolled cellular proliferation and contributes to malignant transformation when accompanied by additional genetic alterations (80).

Focusing on the MAPK/ERK pathway, in AS, mutations are commonly identified in the RAS gene family, specifically KRAS, HRAS, and NRAS isoforms that encode different monomeric GTPases. Most of these mutations are missense mutations (23, 26–29). Other mutations observed in AS concern BRAF, the RAF isoform (26, 27, 29). Mutations in RAS regulatory proteins, such as NF1, have also been implicated in tumorigenesis and drug resistance (23, 26, 27). The prevalence of RAS/RAF/MEK/ERK pathway mutation in AS varies across studies, ranging from no mutation (78, 81) to mutation frequencies as high as 53% (27), depending on the anatomic tumor location (**Supplementary 2**).

In addition to the well-characterized and frequently studied genetic alterations, it is important to note that a substantial number of additional genetic alterations have been identified in AS, albeit at much lower incidence rates. Mutations such as those in ATRX (26, 29), ARID1A (26–29), CRKL (23, 29), ATM (23, 29), ERCC4 (29) occur much less frequently, typically not exceeding 10% in studied population. These low-incidence genetic alterations may still have clinical significance but their rarity poses challenges for large-scale analysis and therapeutic targeting.

### 3.3 Metabolic pathway dysregulation

A defining characteristic of carcinogenesis is the ability of cancer cells to evade apoptosis and maintain continuous proliferation, even in the presence of cellular abnormalities - a process mediated by several metabolic pathways (82). Recent sequencing studies have identified several angiogenic and oncogenic pathways as central drivers of AS development: the VEGF pathway, the angiopoietin-TIE (ANGPT-TIE) pathway, the RAS-RAF-MEK-ERK pathway, and the PI3K/Akt/mTOR pathway (27, 81, 83–85) (**Table 2**; **Supplementary 3**).

**Table 2:** Summarized data regarding metabolic pathway biomarkers in AS.

Pathway	Representative	Number of AS samples	Number of positive AS samples	% positivity (Mean % + Range)	Pattern of expression (Focal/Heterogeneous/Diffuse)	Method	Classification (Diagnostic/Prognostic/Potential therapeutic)	Reference
VEGF pathway	VEGF-A	138	118	86% (85-94)	Heterogeneous	IHC	Potential therapeutic	(31, 83, 89, 90)
	VEGF-C	102	64	63% (12-88)	Heterogeneous	IHC	Potential therapeutic	(89, 90)
	VEGFR-1	68	53	78% (62-94)	Heterogeneous	IHC	Potential therapeutic	(67, 89)
	VEGFR-2	114	96	84% (65-100)	Heterogeneous	IHC	Prognostic Potential therapeutic	(45, 66, 67, 83, 89)
	VEGFR-3	121	102	84% (53-100)	Heterogeneous	IHC	Potential therapeutic	(45, 67, 83, 89, 91, 92)
ANGPT-TIE pathway	ANG1	51	48	94%	Heterogeneous	IHC	Prognostic Potential therapeutic	(85)
	ANG2	50	31	62%	Heterogeneous	IHC	Potential therapeutic	(85)
	TIE1	51	46	90%	Heterogeneous	IHC	–	(85)
	TIE2	51	50	98%	Heterogeneous	IHC	–	(85)
MAPK/ERK pathway	p-ERK1/2	120	65	54% (31-95)	Heterogeneous	IHC	Potential therapeutic	(81, 84, 114)
PI3K/Akt/mTOR pathway	pS6K and/or p-4eBP1	40	17	43%	Diffuse	IHC	–	(81)
	p110α	21	19	90%	Heterogeneous	IHC	Potential therapeutic	(84)
	pAKT	68	58	85%	Heterogeneous	IHC	–	(90)
	p-4eBP1	68	60	88%	Heterogeneous	IHC	–	(90)
	eIF4E	68	59	87%	Heterogeneous	IHC	–	(90)

Detailed data can be found in **Supplementary 3**.

IHC, Immunohistochemistry; VEGF, vascular endothelial growth factor; VEGFR, vascular endothelial growth factor receptor; ANG, angiopoietin; TIE, tyrosine kinase with immunoglobin and EGF homology domains; p-ERK1/2, phosphorylated-extracellular signal-regulated kinases 1/2; pS6K, phosphorylated protein S6 kinase B1; pAKT, phosphorylated protein kinase B; eIF4E, eukaryotic translation initiation factor 4E; p-4eBP1, phosphorylated 4E-binding protein 1.

#### 3.3.1 VEGF pathway

The VEGF and its receptor VEGFR system play pivotal roles in both physiological and pathological angiogenesis (86). VEGF-A, a key angiogenic factor, activates VEGFR-1 (FLT1) and VEGFR2 (KDR), leading to receptor cross-phosphorylation and dimerization. This activation promotes tumor growth by enhancing blood vessel formation and increasing the potential for hematogenous metastases (87). VEGFR-1, a kinase-impaired RTK, can both negatively and positively regulate angiogenesis (88). Conversely, VEGFR-2, a highly active RTK, has critical functions in regulating the proliferation and migration of endothelial cells through distinct signal transduction pathways, including the PI3K/Akt/mTOR and the RAS-RAF-MEK-ERK pathway (86, 88). VEGF-C and VEGF-D primarily regulate lymphangiogenesis through their receptors, VEGFR-2 and VEGFR-3 (FLT4). These factors are essential for the development and function of the lymphatic network, primarily via VEGFR-3, which is predominantly expressed in lymphatic epithelium (68). In many solid tumors, VEGF-C and VEGF-D are thought to contribute to lymphatic metastasis by inducing tumor lymphangiogenesis and directing metastasis to lymph nodes via lymphatic vessels (87).

Immunohistochemical analysis of the VEGF pathway component reveals significant overexpression of several pro-angiogenic factors in AS. VEGF-A expression has been detected in 76% to 94% of AS cases, indicating a consistent role in promoting angiogenesis within these tumors (31, 83, 89, 90). However, the positivity of VEGF-C exhibits heterogeneity, with studies reporting varying frequencies (89, 90). Itakura et al. found only 11.7% of cases positive for VEGF-C immunostaining, whereas Lahat et al. detected positivity in 88.4% of cases. Furthermore, VEGF-C expression varied from weak, focal to heterogeneous, suggesting potential variations in its role or regulation within different AS subtypes (67, 89). Analysis of VEGF receptors demonstrates high expression of VEGFR-1, VEGFR-2, and VEGFR-3 in AS tissue, with mean positivity rates of 78%, 84%, and 84%, respectively (**Table 2**) (45, 66, 67, 83, 89, 91, 92). The pattern of expression varies, with VEGFR-3 typically showing diffuse staining, while VEGFR-1 and VEGFR-2 exhibit more heterogeneous patterns (45, 66, 67, 83, 89, 91, 92).

The prognostic significance of VEGF receptors in AS reveals a paradoxical relationship distinct from other malignancies. While VEGFR-1 and VEGFR-3 expression showed no significant association with clinical outcomes (89), reduced or absent expression of VEGFR-2 has been linked to unfavorable prognosis in AS (67, 89). This contrasts with observation in other cancer types, where elevated VEGFR-2 typically signifies advanced disease (93, 94). Interestingly, previous research has demonstrated a correlation between VEGF-A and VEGFR-2 expression and cell proliferation in scalp and face AS (83), suggesting that preserved VEGFR-2 expression may instead reflect endothelial differentiation and cellular maturation, potentially explaining its inverse prognostic role. Additionally, elevated serum levels of VEGF-A and VEGF-D have been observed with advancing tumor stage in AS patients (95). Together, these results suggest that both VEGF-A/VEGFR-2 and VEGF-C and D/VEGFR-3 signaling axes participate in cell survival and tumor progression via autocrine and paracrine mechanisms within the AS microenvironment (83).

Given the prominent role of the VEGF pathway in AS, therapeutic strategies targeting this pathway have been extensively investigated. However, clinical trials evaluating its targeted therapies have yielded disappointing results (96). Single-agent VEGF inhibitors such as bevacizumab have shown limited efficacy, with a response rate of only 9% and a median progression-free survival (PFS) of 3 months in a phase II study (97, 98). The addition of bevacizumab to paclitaxel did not significantly improve outcomes compared to paclitaxel alone in a randomized phase II trial (98). TKIs targeting VEGFR, such as sorafenib, pazopanib, regorafenib have shown modest but promising activity in AS patients (99–101). A retrospective study of pazopanib in AS patients reported a PFS of 3 months and a median OS of 9.9 months (100). Similarly, another TKI, regorafenib showed some preliminary activity in a small cohort of AS patients, with median PFS and OS of 3.55 and 11.4 months, respectively (101).

#### 3.3.2 ANGPT-TIE pathway

The ANGPT-TIE system plays a role in vascular development, homeostasis, and pathological angiogenesis (102). This system comprises two RTKs, TIE1 and TIE2 (TEK), primarily expressed on endothelial cells, and three corresponding ligands, angiopoietin-1 (ANG1), angiopoietin-2 (ANG2), and angiopoietin-4 (ANG4). While ANG proteins serve as ligands for TIE2, TIE1 lacks a known ligand and is thought to be activated via its interaction with TIE2 (103). Among the angiopoietin, ANG1 and ANG2 have been the primary focus of research, whereas the function of ANG4 remains less well characterized. ANG-1 functions as a TIE2 agonist, supporting endothelial cell survival, vascular stability, and endothelial barrier integrity (104). TIE2 activation by ANG1 leads to downstream signaling through the PI3K/Akt/mTOR pathway (105). In contrast, ANG-2 can function as both an agonist and antagonist of TIE2, depending on the context, and can inhibit the ANG1-TIE2 signaling axis (106). ANG2 appears to play a pivotal role in vascular remodeling and angiogenesis (106). Under physiological conditions, ANG1-TIE2 signaling maintains vascular quiescence by reinforcing endothelial cell barrier function and suppressing inflammatory responses. However, during pathological conditions such as inflammation or tumor angiogenesis, increased ANG2 levels can destabilize blood vessels and promote vascular permeability (107, 108).

In the context of AS, the expression and functional role of the ANGPT-TIE pathway remain incompletely understood. However, Buehler et al. demonstrated that key components of this system are frequently expressed in AS, with ANG1, TIE1, and TIE2 detected in most cases, while ANG2 expression was observed in 42% of tumors (85). Notably, higher ANG1 expression was associated with improved survival in AS patients (85). In a separate study, serum ANG2 levels were significantly increased in 11 face and scalp AS patients compared to the healthy control, with ANG2 levels further increasing in advanced-stage AS patients (109). Corroborating these findings, ANG2 mRNA expression was upregulated in AS relative to other soft tissue sarcomas (65). The differential expression and prognostic implications of ANG1 and ANG2 in AS suggest different roles and modalities in disease progression. ANG2, which can function as both agonist and antagonist of TIE2, appears to play a more prominent role in AS pathogenesis (65, 109).

From a therapeutic perspective, targeting the ANGPT-TIE pathway has shown promise in preclinical models. Recent studies have reported the efficacy of TIE2 inhibitor therapy in inhibiting AS growth in murine models of this disease (110). However, clinical translation has proven challenging. A phase II study of trebananib, a peptibody targeting both ANG1 and ANG2, failed to demonstrate responses in AS patients (111). Collectively, these findings suggest that the ANGPT-TIE system may be considered as a prognostic and therapeutic target in this aggressive vascular malignancy. However, further research is needed to elucidate the precise mechanism by which this pathway contributes to AS pathogenesis and to develop more effective targeted therapies.

#### 3.3.3 RAS-RAF-MEK-ERK pathway (MAPK/ERK pathway)

The RAS-RAF-MEK-ERK pathway consists of an intracellular signaling cascade for cell proliferation, differentiation, and survival (69). The signaling cascade is initiated by the binding of extracellular ligands to cell surface receptors, such as RTKs and GPCRs (69). This binding activates RAS proteins through the exchange of GDP for GTP. Activated RAS subsequently recruits and activates RAF kinases, which in turn phosphorylate and activate MEK1/2. MEK1/2 then phosphorylates and activates ERK1/2, the final kinases in the cascade (69, 112). After RAF-MEK-ERK activation, phosphorylated ERK1/2 (p-ERK1/2) translocates to the nucleus and phosphorylates numerous substrates, including transcription factors, thereby modulating gene expression and influencing cell behavior (113). The MAPK/ERK pathway is tightly regulated under normal physiological conditions. However, metabolic alterations in components of this pathway are prominently associated with carcinogenesis (69).

In the context of AS, activation of the RAS-RAF-MEK-ERK pathway has been demonstrated via the p-ERK1/2 immunostaining. The prevalence of p-ERK1/2 positivity in clinical AS samples varies considerably across studies, ranging from 31% to 95%. Furthermore, the labeling intensity exhibits heterogeneity, with patterns ranging from weak and focal to strong and diffuse (**Table 2**) (81, 84, 114). These findings are consistent with the observations of Chadwick et al., who reported p-ERK1/2 activation in all tumors of vascular origin (115). The wide range of p-ERK1/2 positivity and staining patterns observed in AS suggest a complex and potentially heterogeneous role for MAPK/ERK signaling in AS pathogenesis. Understanding the complex regulation and cross-talk of the RAS-RAF-MEK-ERK pathway with other signaling networks is crucial for developing more effective targeted therapies and overcoming drug resistance in AS treatment (80).

#### 3.3.4 PI3K/AkT/mTOR pathway

The PI3K/Akt/mTOR signaling pathways play essential roles in regulating cellular growth and survival under physiological and pathological conditions (73, 116). Dysregulation of the PI3K/Akt/mTOR pathway promotes aberrant proliferative signaling and disrupts cellular metabolic homeostasis, which are hallmarks of cancer (117). The core components of this pathway include PI3K, AKT (protein kinase B), and mTOR. PI3K is a heterodimeric enzymen composed of a catalytic subunit (p110α) and an adaptor/regulatory subunit (p85α). It catalyzes the phosphorylation of phosphatidylinositol-4,5-bisphosphate (PIP2) to phosphatidylinositol-3,4,5-triphosphate (PIP3), which functions as a key secondary messenger (73, 116). This event facilitates Akt recruitment to the plasma membrane, where it undergoes phosphorylation at T308 and S473 residues (116). mTOR is a serine/threonine protein kinase acting downstream of PI3K and Akt, functions within two distinct complexes: mTORC1 and mTORC2, each regulating distinct cellular processes (73). mTORC1 controls protein synthesis and cellular growth by phosphorylating downstream translation effectors. These include the eukaryotic translation initiation factor 4E (eIF4E)-binding protein 1 (4E-BP1) and the ribosomal protein S6 kinase B1 (S6K1). Upon phosphorylation, S6K1 enhances mRNA translation, while phosphorylation of 4E-BP1 releases its inhibitory effect on eIF4E, which is essential for cap-dependent translation initiation (117). mTORC2 regulates cell proliferation and survival through phosphorylation of Akt at Ser473 (117, 118). mTOR activity is modulated by a complex network of upstream modulators, encompassing both positive and negative regulators. Growth factors and their receptors, such as VEGFRs and their ligands, serve as positive regulators by transmitting signals to mTOR via the PI3K/Akt pathway (73). Conversely, the tumor suppressor phosphatase and tensin homolog (PTEN) acts as a critical negative regulator of mTOR activity. PTEN antagonizes PI3K activity by dephosphorylating PIP3, thereby attenuating Akt activation and subsequent mTOR signaling. Loss of PTEN function results in constitutive PI3K/Akt pathway activation, often observed in various cancers (73).

Recent studies have confirmed that alterations of the PI3K/Akt/mTOR pathway are a key oncogenic mechanism driving AS development (81, 84, 90). Multiple investigations have examined different components of this pathway in AS, revealing its widespread activation and potential role in disease progression (**Table 2**). Italiano et al. reported that 42% of cases were positive for p-S6K and p-4E-BP1, two classical downstream targets of mTORC1 (81). Wan et al. focused on the expression of catalytic subunit p110α of PI3K, finding that it was exclusively detected in the cytoplasm of 90.5% of AS cases (84). Additionally, Lahat et al. examined pAkt, p4E-BP1, and eIF4E, reporting a high prevalence of positivity in 85%, 88%, and 87% of AS cases, respectively (90). Notably, p-4E-BP1 expression intensity was significantly higher in metastatic AS compared with localized lesions, suggesting its potential role in the metastatic progression of AS (90). Several studies focused on the down-regulation of PTEN, a negative regulator of the PI3K/Akt/mTOR pathway (78, 84). Verbeke et al. reported decreased PTEN expression in 41% of bone AS compared to soft tissue AS (78). In agreement, Wan et al. observed PTEN downregulation in scalp and face AS compared to hemangiomas (84). These findings collectively indicate that the PI3K/Akt/mTOR pathway is frequently activated in AS through various mechanisms, including increased expression of downstream effectors and downregulation of negative regulators.

Activation of the PI3K/Akt/mTOR pathway in AS provides a strong rationale for targeting this pathway with selective inhibitors. Currently, two FDA-approved PI3K inhibitors, alpelisib and copanlisib, are available for the treatment of various cancers (119, 120). Alpelisib is a specific PI3K inhibitor that selectively targets p110α (119). Its efficacy has been demonstrated in preclinical mouse models (121) and PIK3CA-mutant breast carcinoma patients (79), suggesting its potential as a targeted therapy for primary breast AS. Copanlisib, a pan-class I PI3K inhibitor with activity against all four isoforms, has shown efficacy against solid tumors and hematological malignancies (120). An alternative therapeutic strategy involves targeting mTOR. mTOR inhibitors, including rapamycin (sirolimus) and its analogs temsirolimus and everolimus, suppress mTOR activity. Everolimus has shown efficacy in breast cancer by inhibiting cell growth through downregulation of the PI3K/Akt/mTOR signaling pathway, highlighting its potential as a therapeutic option for primary breast AS (122).

In general, targeted monotherapies modulating individual angiogenesis pathways in AS have demonstrated limited efficacy in clinical studies, with response rates generally below 10% (97, 100). Instead, combination therapeutic strategies targeting multiple parallel signaling pathways have emerged as more promising approaches in preclinical studies. In a mouse model of AS, combined inhibition of mTOR (rapamycin) and MEK (trametinib) led to sustained tumor regression compared to monotherapy with either agent alone (115). Furthermore, preclinical studies have demonstrated that dual inhibition of VEGF and MAPK pathway using cediranib (VEGFR inhibitor) and trametinib (MEK inhibitor) had additive effects *in vitro* and combinatorial effects *in vivo*, reducing AS cell survival (114). These findings were corroborated by RNA sequencing analysis, which revealed distinct expression signatures between tumors treated with trametinib alone and those treated with both trametinib and cediranib (114). The synergistic effects observed in these preclinical studies provide a strong rationale for further investigation of combination therapies in AS.

### 3.4 Characteristic proteins

Protein expression, detectable through immunohistochemistry (IHC), offers a readily accessible approach to evaluating tumor characteristics and identifying potential therapeutic targets. While genetic and metabolomics analyses provide valuable insights into the underlying biology of AS, protein biomarkers often serve as the initial diagnostic and prognostic indicators in clinical practice, guiding treatment decisions and risk stratification. In the following section, we will integrate and summarize existing data surrounding the key protein biomarkers in AS, including their diagnostic utility, prognostic significance, and potential as therapeutic targets (**Table 3**; **Supplementary 4**).

**Table 3:** Summarized data regarding protein biomarkers in AS.

Protein	Location (Nucleus/ Cytoplasm/Membranous)	Number of AS samples	Number of positive AS samples	% Positivity (Mean % + Range)	Pattern of expression (Focal/ Heterogeneous/Diffuse)	Cut-off threshold (%)	Method	Classification (Diagnostic/ Prognostic/Potential therapeutic)	Reference
p53	Nucleus	283	133	47% (6-100)	Heterogeneous	>20-40%	IHC	Prognostic	(31, 52, 66, 78, 81, 84, 129–132)
Ki-67	Nucleus	194	132	68% (33-100)	Heterogeneous	>10-33%	IHC	Diagnostic Prognostic	(83, 91, 92, 129, 130, 140–144)
ERG	Nucleus	278	268	96% (22-100)	Heterogeneous	NA	IHC	Diagnostic	(52, 141, 154–161, 246)
FLI-1	Nucleus	92	89	97% (75-100)	Diffuse	NA	IHC	–	(129, 142, 157–160, 167)
c-MYC	Nucleus	588	254	43% (22-90)	Heterogeneous	>5-50%	IHC	Diagnostic Prognostic	(43–46, 49–52, 132, 140, 156, 171, 173)
vWF (FVIII-RA)	Cytoplasm	269	210	78% (11-100)	Heterogeneous	NA	IHC	–	(66, 83, 91, 92, 130, 131, 141, 142, 167, 180–182, 200, 225, 247–249)
Vimentin	Cytoplasm	61	60	98% (98-100)	Heterogeneous	NA	IHC	–	(91, 130, 131, 142, 189)
CD31	Membranous	846	767	91% (33-100)	Heterogeneous	NA	IHC	–	(44, 52, 66, 83, 90–92, 129–132, 141, 142, 154–158, 160, 167, 180, 182, 189, 199, 200, 215, 225, 246–255)
CD34	Membranous	728	493	68% (13-100)	Heterogeneous	NA	IHC	–	(52, 66, 83, 91, 92, 129–131, 141, 142, 154–158, 160, 167, 180–182, 189, 200, 215, 225, 246–248, 250–255)
CD117 (c-Kit)	Membranous	302	109	36% (0-67)	Heterogeneous	NA	IHC	Potential therapeutic	(66, 67, 78, 90, 129, 132, 141, 210, 255)
Podoplanin	Membranous	377	230	61% (25-100)	Heterogeneous	NA	IHC	–	(44, 52, 83, 90, 91, 132, 154, 161, 215, 216, 250)
Claudin-5	Membranous	136	132	97% (97-100)	Diffuse	NA	IHC	–	(220, 221)
UEA-I	Membranous	74	60	81% (27-100)	Heterogeneous	NA	IHC	–	(181, 182, 200, 225, 247)
PD-L1	Membranous	634	235	37% (19-100)	Heterogeneous	>1-10%	IHC	Prognostic Potential therapeutic	(23, 140, 144, 215, 232–236, 256)

Detailed data can be found in **Supplementary 4**.

IHC, Immunohistochemistry; ERG, ETS-related gene; FLI-1, Friend leukemia integration 1; FVIII-RA, Factor VIII-related antigen; UEA-I, Ulex europaeus agglutinin I; PD-L1, Programmed-death ligand 1.

#### 3.4.1 p53

The tumor suppressor protein p53, encoded by the TP53 gene, is a critical regulator of genomic stability, preventing the accumulation of oncogenic mutations that lead to malignant tumors. Frequently termed the “guardian of the genome”, p53 is activated in response to various cellular stresses, including hypoxia, oncogene activation, DNA damage, and nucleotide deprivation (123). Upon activation, p53 modulates the transcription of numerous target genes involved in key cellular processes, including apoptosis, cell cycle arrest, DNA repair, and cellular senescence (24). Missense mutations in TP53 lead to the overexpression of mutant p53 (mutp53). Unlike wild-type p53 (wtp53), which exerts tumor-suppressive functions, mutp53 not only loses these protective capabilities but can even acquire oncogenic gain-of-function activities, promoting tumor progression, metastasis, and chemo-resistance (124, 125).

The wtp53 protein has a short half-life under normal physiological conditions and is typically undetectable by IHC (126). However, in response to genotoxic stress, p53 protein levels rapidly increase due to post-transcriptional stabilization mechanisms (126, 127). In contrast to wtp53 protein, mutp53 exhibits increased stability and can be detected in the nuclei of neoplastic cells (128). This difference in stability has led to the use of p53 IHC as a surrogate marker to predict the presence of mutp53, based on the premise that only the stabilized (presumably mutated) protein is detectable. However, the interpretation of p53 immunohistochemical studies remains controversial and lacks standardization. Some investigations consider cases to be immunoreactive based on staining intensity and the percentage of positive cells (66, 78, 84, 129), while others apply different cut-off thresholds (52, 81, 130–132). This variability in interpretation can lead to discrepancies in results and difficulties in comparing AS studies.

In the context of AS, immunohistochemical analyses have detected p53 overexpression from 6% to 100% of AS cases(**Table 3**). Importantly, this overexpression has been associated with worse disease-free survival (DFS), with p53-positive cases (defined as >20% nuclear positivity) showing a median DFS of 3.4 months compared to 14.9 months for p53-negative cases (81). Studies have also reported significantly higher p53 immunoreactivity in the scalp and face AS compared to benign hemangiomas (84). Interestingly, p53 protein accumulation does not always correlate with TP53 gene mutations in AS, suggesting that p53 overexpression may result from specific oncogenic stresses leading to wtp53 stabilization, rather than exclusively from mutations (31, 81). Furthermore, elevated expression of p53 protein has been found to correlate with increased VEGF expression in nearly 80% of AS cases examined, indicating a potential interplay between p53 and the angiogenic pathway in AS pathogenesis (31). These findings collectively highlight the importance of p53 protein expression in AS pathogenesis and its potential as a prognostic biomarker. However, the lack of standardization in immunohistochemical interpretation and cut-off values remains a challenge in accurately assessing the p53 status in these tumors.

Despite its widespread mutation across cancers, targeting mutp53 therapeutically has proven challenging. However, a recent investigation into small molecules capable of reactivating mutp53 has yielded promising results (133). Notably, APR-246 (eprenatapopt), a first-in-class p53 reactivator, has demonstrated the ability to refold mutp53 and restore its function, leading to the induction of p53 target gene expression. APR-246 has shown clinical activity, particularly in myeloid malignancies (133), and can be explored further for broader application in AS.

#### 3.4.2 Ki-67

The nuclear protein Ki-67, encoded by the MKI67 gene, is a widely recognized marker of cellular proliferation and serves as a marker for assessing cell division in cancer research and clinical settings. Ki-67 is expressed during all active phases of the cell cycle (G1, S, G2, and M phases) but is absent in quiescent cells in the G0 phase. Functionally, it is involved in critical processes such as ribosomal RNA synthesis, heterochromatin organization, and the formation of the perichromosomal layer during mitosis (134). In cancer, Ki-67 expression is strongly associated with tumor proliferation and serves as a prognostic indicator in various malignancies, including breast, prostate, lung, and soft tissue tumors (135–138). High Ki-67 levels generally indicate more aggressive tumors and correlate with poor prognosis, larger tumor size, lymphatic invasion, and metastases (134, 139).

In the context of AS, immunohistochemical studies have consistently demonstrated high expression of Ki-67, with a mean positivity of 68% across AS cases (**Table 3**) (83, 91, 92, 129, 130, 140–144). Notably, several studies have reported 100% Ki-67 positivity in AS cases, regardless of the anatomic location (91, 129, 141–143). This high proliferative index distinguishes AS from benign vascular lesions, such as hemangioma, making Ki-67 a valuable diagnostic tool (83, 92, 129, 143). A study of breast vascular lesions established a Ki-67 index cutoff of 175, which demonstrated 90% sensitivity and 95% specificity in differentiating AS from HA. Furthermore, Ki-67 expression correlates with the clinical course of cutaneous AS, with patients exhibiting strong Ki-67 expression experiencing more unfavorable outcomes (91). The high proliferative activity indicated by elevated Ki-67 levels in AS is associated with increased rates of metastases and mortality (145). These findings collectively suggest that Ki-67 expression not only aids in the diagnosis of AS but also serves as a prognostic marker, potentially guiding treatment decisions and risk stratification for patients with this aggressive vascular malignancy.

#### 3.4.3 ERG

ERG (ETS-related gene) is a transcription factor belonging to the ETS (erythroblast transformation-specific) family, which plays crucial roles in embryonic development, cell proliferation, differentiation, inflammation, angiogenesis, and apoptosis (146, 147). As a nuclear protein, ERG binds to purine-rich DNA sequences and is critical for maintaining vascular integrity, hematopoietic stem cell (HSCs) function, and endothelial homeostasis (148, 149). Beyond its normal physiological roles, ERG has been implicated in the pathogenesis of various malignancies (150–152). In prostate cancer, ERG has been shown to repress the transcription of the tumor suppressor PTEN, potentially activating the PI3K/Akt pathway and increasing angiogenesis, invasion, and metastasis (153).

In AS, sequencing studies have demonstrated ERG exceptional sensitivity, with strong and diffuse nuclear staining observed in 100% of AS cases, irrespective of anatomic location (52, 141, 154–160). ERG essential roles in regulating angiogenesis, endothelial cell differentiation, and apoptosis may contribute to its consistent expression in AS (141). However, ERG expression is not exclusive to AS as nuclear ERG staining has been observed in various benign or malignant vascular tumors, including hemangioma, lymphangiomas, epithelioid hemangioendotheliomas, and Kaposi sarcoma (141, 150, 161). Among non-endothelial neoplasms, ERG expression has been reported in prostatic adenocarcinomas, Ewing sarcomas, and AML (141, 150–152, 159). Despite these limitations, ERG remains a highly specific marker for vascular neoplasms in general. This high specificity, combined with its superior sensitivity, positions ERG as a valuable diagnostic tool for vascular tumors, particularly when used with other markers and histological examination. In the context of AS diagnosis, its consistent and strong expression pattern provides significant diagnostic utility, especially in challenging cases or when dealing with limited biopsy material. Unfortunately, therapeutic strategies exploiting ERG overexpression in AS remain largely unexplored.

#### 3.4.4 FLI-1

Friend leukemia integration 1 is a transcription factor belonging to the ETS family, characterized by its conserved ETS DNA-binding domain. FLI-1 plays critical roles in normal hematopoiesis, endothelial cell survival, and vascular development (162). By regulating genes associated with cell proliferation, differentiation, and survival, FLI-1 is essential for maintaining HSCs and their differentiation into mature blood cells (163). FLI-1 is also implicated in pathological processes, as its dysregulation contributes to the development of various malignancies, including Ewing’s sarcoma, erythroleukemia, B-cell lymphomas, and AS (164–166). Its role in promoting angiogenesis and tumor progression further underscores its importance in AS pathogenesis.

In the context of AS, multiple studies have demonstrated 100% sensitivity of FLI-1 with strong and intense nuclear staining (129, 157–160, 167). The nuclear localization of FLI-1 staining offers a distinct advantage over traditional cytoplasmic or membranous endothelial markers, reducing artifacts associated with endogenous peroxidases or biotin (168). However, it is important to note that while FLI-1 is a sensitive marker for AS, its expression has also been detected in benign vascular tumors, such as hemangiomas (129), or other non-endothelial neoplasms, such as Ewing’s sarcoma, erythroleukemia, lymphoma (164–166). McKay et al. reported 100% sensitivity for AS but only 29% specificity, as FLI-1 expression was detected in other tumor types, including squamous cell carcinomas, melanomas, atypical fibroxanthomas, and leiomyosarcomas (159). Consequently, its expression in other tumor types necessitates the use of additional specific markers or molecular confirmation for the definitive diagnosis of AS. So far, no FLI-1-targeted therapies have been specifically explored for AS treatment.

#### 3.4.5 c-MYC

c-MYC transcription factor, a member of the MYC proto-oncogene family, is a nuclear phosphoprotein that regulates key cellular processes including proliferation, differentiation, and apoptosis (169). Importantly, c-MYC is also known to stimulate angiogenesis and may promote invasion and metastasis (170). c-MYC functions as a transcriptional regulator by forming heterodimers with its obligatory partner MAX (Myc-associated factor-X) and binding to enhance box sequences (E-boxes) in target gene promoters (38, 169). As a transcriptional amplifier, c-MYC enhances the expression of actively transcribed genes by recruiting histone acetyltransferases, promoting transcription elongation through P-TEFb recruitment, and regulating CDK9 SUMOylation (169). Dysregulation of c-MYC expression, often through gene amplification or chromosomal translocation, is implicated in numerous human cancers, underscoring its significance as both a critical regulator of normal cellular processes and a potent oncogene (39–41).

In AS, c-MYC overexpression has been observed with varying frequencies depending on the tumor etiology. Studies have shown that 54-100% of radiotherapy-associated AS exhibit high-level c-MYC protein overexpression (43–46, 49, 51, 132, 156, 171), with the majority of radiotherapy-associated AS cases characterized as high-grade tumors (46, 171). c-MYC overexpression has also been reported in Stewart-Treves AS (49, 171). While c-MYC overexpression is most prevalent in sAS, it has also been observed in pAS (49–52, 132, 140, 156). Hogeboom-Gimeno et al. reported positive c-MYC staining in 39.5% of pAS cases across multiple anatomic sites, including the breast, skin, soft tissue, and visceral location (49). In primary cutaneous AS, c-MYC overexpression has been observed in a subset of cases, with elevated expression correlating significantly with higher-grade tumors (50). Importantly, c-MYC has not been reported in atypical vascular lesions, making c-MYC analysis a crucial diagnostic tool for distinguishing AS from other vascular lesions (45, 46, 51, 171). Prognostically, AS patients displaying c-MYC protein overexpression have significantly reduced OS compared to those without overexpression (49, 171). These findings highlight the complex role of c-MYC in AS pathogenesis and its potential utility as a diagnostic and prognostic marker. The variable expression patterns observed across different AS subtypes suggest that c-MYC may play distinct roles in the development and progression of pAS and sAS.

MYC, a frequently amplified proto-oncogene, demonstrates a strong correlation with c-MYC protein overexpression. Sequencing studies have shown a consistent concordance between MYC protein expression measured by IHC and gene amplification assessed by Fluorescence *In Situ* Hybridization (FISH). Specifically, excellent FISH and IHC concordance has been observed in both primary and secondary mammary AS (43, 45, 46, 49, 51). However, for non-mammary sites, concordance between FISH and IHC was generally poor (49–52). These findings reaffirm the utility of MYC FISH and IHC as diagnostic tools in breast AS while suggesting limited applicability as general surrogate markers in AS from other anatomical locations. The discordance between MYC gene amplification and protein overexpression in non-mammary locations indicates alternative mechanisms for protein upregulation, such as altered mRNA stability, enhanced transcription/translation, or epigenetic modifications (172). Additionally, inconsistencies in IHC interpretation, including non-specific immunostaining or variable positivity thresholds, may contribute to this discrepancy. Indeed, the interpretation of c-MYC positivity varies among investigations, with some studies evaluating positivity based on staining intensity (46, 49, 50, 156, 171), while others employ different cut-off values (51, 52, 132, 140, 173). These findings emphasize the importance of considering anatomical location when interpreting MYC expression data in AS, as the underlying mechanisms of MYC dysregulation may differ between mammary and non-mammary sites. The recent phase I clinical trial results of a MYC inhibitor demonstrating safety and anti-tumor activity in solid tumors (174) suggest that c-MYC-targeted therapies may become available for AS patients shortly.

#### 3.4.6 Von Willebrand factor

Factor VIII (FVIII) and Factor VIII-related antigen (also known as von Willebrand factor; vWF) are distinct but closely associated glycoproteins that play crucial roles in hemostasis (175, 176). FVIII, encoded by the F8 gene, is an essential coagulation factor that circulates in plasma bound to vWF (176, 177). This binding is critical for FVIII stability, as it degrades rapidly when not bound to vWF. vWF, beyond its role as a carrier protein for FVIII, promotes platelet adhesion and aggregation at sites of vascular injury (175). It is synthesized in endothelial cells and megakaryocytes and undergoes complex post-translational modifications that influence its affinity for FVIII (178). Upon endothelial cell activation, vWF is rapidly secreted from Weibel-Palade bodies, functioning as an acute phase protein with multifaceted roles in vascular inflammation (176). Immunohistochemical detection for vWF is widely used as a marker for endothelial differentiation in diagnostic pathology, particularly in vascular tumors such as AS (179).

In AS, vWF typically shows a granular to homogeneous cytoplasmic staining pattern in neoplastic endothelial cells (66, 83, 130, 141, 180, 181). However, its expression can be variable, with well-differentiated tumors exhibiting more consistent staining compared to poorly differentiated ones (182). Studies have reported a mean positivity of immunostaining for vWF of 78% across AS cases (**Table 3**). vWF expression in AS often shows weak and focal staining (66, 83, 130, 180). While vWF remains a valuable tool in the diagnosis of AS, it is frequently used in conjunction with other endothelial markers such as CD31, CD34, ERG, and VEGFR-2 for optimal diagnostic accuracy (17, 66). This panel approach is necessary because vWF, being a specific marker for endothelial differentiation, is also expressed in other vascular lesions, including hemangiomas (66, 83).

#### 3.4.7 Vimentin

Vimentin, a type III intermediate filament protein encoded by the VIM gene in humans, is a key structural component of the cytoskeleton in mesenchymal cells. It plays a crucial role in preserving cytoplasmic integrity, maintaining cell shape, and stabilizing cytoskeletal interaction (183, 184). Vimentin participates in diverse cellular processes, including cell migration, adhesion, and signal transduction (185). Its expression is frequently used as a marker for epithelial-to-mesenchymal transition (EMT), a process critical for cancer progression and metastasis (186). Overexpression of vimentin has been associated with various malignancies, such as lung and gastric cancers, where it is associated with increased metastatic potential, higher nuclear grade, and poorer overall survival outcomes (187, 188).

In AS, vimentin expression is commonly observed with positivity up to 100% (91, 130, 131, 142, 189). While vimentin is generally associated with AS, its specificity is limited as it may also be expressed in carcinomas (186). Studies have shown that vimentin contributes to tumor progression by mediating cytoskeleton architecture and maintaining intracellular mechanical homeostasis (190). The role of vimentin in cancer extends beyond its structural function, as it is involved in regulating autophagy, intracellular signaling pathways, and protecting cells from caspase-induced proteolysis (183). Notably, it has been identified as a downstream effector of the PI3K/Akt signaling pathway, where its phosphorylation enhances cellular migration (191). These findings highlight vimentin’s potential as a therapeutic target in cancer, including AS, where its expression may contribute to the aggressive nature of the disease.

#### 3.4.8 CD31

CD31, also referred to as platelet endothelial cell adhesion molecule-1 (PECAM-1), is a 130 kDa transmembrane glycoprotein encoded by the PECAM1 gene. CD31 is predominantly expressed in endothelial cells (192), platelets (193), various leukocyte subpopulations (194), and hematopoietic progenitor cells (195). It plays crucial roles in cellular immunity and vascular biology, including cell adhesion, transendothelial migration of leukocytes, angiogenesis, and maintenance of vascular barrier integrity (196). In various malignancies, CD31 has been implicated in promoting tumor cell invasion and metastasis. For example, it has been shown to facilitate metastasis by inducing EMT in hepatocellular carcinoma through upregulation of integrin β1 via the FAK/Akt pathway (197). Furthermore, a high level of CD31 expression combined with high VEGF expression correlated with poor survival in early-stage laryngeal squamous cell carcinoma (198).

In AS, CD31 exhibits strong constitutive expression, with a mean positivity of 91% in AS cases, indicating its importance in tumor development (**Table 3**). The staining intensity is mostly strong and diffuse (44, 66, 129, 154, 156, 167, 189, 199, 200). However, the biological function of CD31 in AS is unclear. Venkataramani et al. have found that most AS contain a small population of CD31-low cells that exhibit increased tumorigenicity and chemoresistance due to more efficient reactive oxygen species (ROS) detoxification (201). These CD31-low cells show nuclear accumulation of Yes-associated protein (YAP), leading to the induction of antioxidative enzymes. The down-regulation of CD31 in AS cells results in loss of endothelial properties and increased resistance to oxidative stress and DNA damage. This mechanism has been linked to intensified YAP signaling, suggesting that the Hippo pathway plays a crucial role in AS progression and chemoresistance (201). However, it is important to note that CD31 is also expressed in other types of vascular tumors, including hemangiomas (66, 83, 129), atypical vascular lesions (44), and epithelioid hemangioendothelioma (141), which may lead to potential diagnostic pitfalls (202). The complex role of CD31 in AS progression and its association with the Hippo pathway suggest potential therapeutic strategies targeting the CD31-YAP signaling axis.

#### 3.4.9 CD34

CD34, a member of the sialomucin family, is a transmembrane phosphoglycoprotein (203). CD34 is expressed across diverse cell populations, including hematopoietic stem/progenitor cells, vascular endothelial cells, and certain mesenchymal cells (204, 205). While its exact function remains elusive, CD34 plays crucial roles in cell adhesion, migration, and signal transduction (203). It facilitates the adhesion of HSCs to the bone marrow extracellular matrix or stromal cells, while also promoting lymphocyte binding to specialized vascular endothelium within lymphoid tissues. CD34 is also involved in maintaining the undifferentiated state of stem cells by promoting proliferation and inhibiting differentiation. Additionally, it contributes to cellular migrations during tissue repair and angiogenesis (203).

In the context of AS, most studies used CD34 as one of the diagnosis markers for AS, albeit with variable positivity. CD34 is expressed in approximately 68% of AS cases (**Table 3**), indicating lower positivity compared to other endothelial markers in AS such as CD31 and ERG (155). The staining intensity ranges from weak and focal to strong and diffuse (**Supplementary 4**). As CD34 is also widely expressed in other vascular tumors (66, 83, 129, 141) and non-vascular cancer cell types (e.g., fibroblastic tumors, gastrointestinal stroma tumors, dermatofibrosarcoma) (206, 207), positivity for CD34 alone does not confirm AS diagnosis. These findings underscore the importance of using a comprehensive immunohistochemical panel including multiple endothelial markers for accurate diagnosis of AS. Interestingly, CD34 co-expression with CD31 is observed in most AS cases (66, 83, 129, 155).

#### 3.4.10 CD117

CD117 (c-Kit) is a transmembrane protein encoded by the KIT gene (208). This 145 kDa protein, comprising 976 amino acids, belongs to the type III RTK family. CD117 is expressed in several cell types, including hematopoietic stem/progenitor cells, mast cells, and certain cancer cells (209). Upon binding to its ligand, stem cell factor (SCF), CD117 forms a homodimer and undergoes autophosphorylation, activating multiple downstream signaling cascades (209). These pathways, including MAPK and PI3K/Akt, regulate critical cellular processes such as survival, proliferation, differentiation, and migration.

CD117 expression has been identified in a subset of AS, with studies reporting positivity in more than 50% of cases (66, 90, 132, 210). Interestingly, benign vascular tumors including hemangiomas and normal adult vessels are negative for CD117 (66, 90, 210, 211). CD117 expression in AS is thought to represent oncofetal expression, where tumor cells revert to a phenotype resembling fetal endothelial cells that exhibit KIT positivity (210). Studies have shown that CD117 is expressed in approximately 90% of soft tissue AS, compared to only 17% in bone AS (78). This differential expression suggests that TKIs targeting CD117 may be more effective in soft tissue AS. Unlike gastrointestinal stromal tumors (GISTs), where activating KIT mutations are common, mutations in the juxtamembrane or tyrosine kinase domains of KIT have not been identified in AS (210, 212, 213). This suggests that CD117 expression in AS is not driven by genetic mutations but rather reflects aberrant protein expression associated with tumorigenesis. There is also evidence of CD117 overexpression contributing to the activation of the PI3K/Akt pathway in soft tissue AS (78).

While CD117 can aid in the diagnosis of AS, its utility is limited due to its expression in other neoplasms, including GISTs and certain sarcoma (212). Therefore, CD117 should be used as part of an immunohistochemical panel alongside other endothelial markers such as CD31 and ERG to improve diagnostic accuracy. From a therapeutic perspective, there is a single case report of a good response to imatinib, a specific TKI, in a soft tissue AS patient, suggesting the need for further research into CD117-targeted approaches to improve outcomes in AS patients (213).

#### 3.4.11 PDPN

Podoplanin is a type-I transmembrane mucin-like glycoprotein encoded by the PDPN gene in humans (214). PDPN is well-conserved across species and serves as a specific marker for lymphatic endothelial cells (214). PDPN plays crucial roles in organ development, cell motility, tumorigenesis, and metastasis (214). It interacts with several proteins, most notably C-type lectin-like receptor 2 (CLEC-2) on platelets, which is essential for proper blood and lymphatic vessel separation during embryonic development (214). PDPN also binds to ezrin and moesin, connecting it to the actin cytoskeleton and influencing cell migration and adhesion (214). Additionally, PDPN modulates the activities of Rho-family GTPases, particularly RhoA, which contributes to the pro-migratory phenotype of PDPN-expressing cancer cells (214).

In AS, PDPN expression is commonly observed with positivity up to 100%, suggesting phenotypic features of lymphatic endothelium (**Table 3**). PDPN exhibits heterogeneous staining intensity, varying from focal to diffuse expression (44, 83, 215, 216). Interestingly, most tumor cells in AS co-expressed PDPN and markers of blood vessel phenotypes (e.g., CD31, ERG, vWF), an unusual combination in normal vessels, suggesting their potential derivation from a common precursor of lymphatic and blood vascular endothelium (52, 91, 216). This co-expression pattern distinguishes AS from hemangiomas, which is consistently negative for PDPN (83, 161, 216). However, it is important to note that PDPN is also expressed in other vascular tumors, including benign lymphangiomas, atypical vascular lesions, and Kaposi sarcoma (44, 216).

#### 3.4.12 Claudin-5

Claudin-5 is a tight junction protein that plays a fundamental role in regulating paracellular permeability in the blood-brain barrier (BBB) (217). This transmembrane protein is encoded by the CLDN5 gene and belongs to the claudin family. It is primarily expressed in endothelial cells during tumor angiogenesis and has emerged as a significant marker in various carcinomas, particularly those of the lung (218, 219). However, data on its expression and functional significance in vascular tumors, including AS, remain limited.

In AS, claudin-5 has demonstrated remarkable sensitivity as an immunohistochemical marker. Studies have shown that 96% to 100% of AS express claudin-5, with strong and uniform staining throughout AS tumor, regardless of differentiation status (220, 221). This high sensitivity makes claudin-5 a promising diagnostic tool for AS, potentially surpassing traditional markers such as vWF in detecting endothelial differentiation in less-differentiated cases (220). Claudin-5 positivity is observed in both vasoformative and solid areas of the tumor, with most cases showing positivity in more than 50% of tumor cells, often approaching 100% (220). However, it is important to note that while claudin-5 demonstrates high sensitivity for AS, its specificity is limited. Claudin-5 has been observed in various carcinomas and other vascular tumors, such as hemangiomas and hemangioendotheliomas (220).

#### 3.4.13 UEA-I

Ulex europaeus agglutinin I is a lectin that has emerged as a valuable marker for vascular endothelial cells and tumors of endothelial origin (222, 223). Studies have shown that UEA-I is more sensitive in detecting endothelial cells compared to traditional markers like vWF (223, 224). In the context of AS, UEA-I has proven to be a valuable diagnostic tool, with a positivity of up to 100% (181, 182, 200, 225). This sensitivity is particularly important in cases where traditional markers, such as CD34, vWF, may yield negative results, especially in less differentiated tumors (182). Therefore, the use of UEA-I in combination with other endothelial markers significantly improves the diagnostic accuracy for AS. The combined use of these markers also helps differentiate AS from other malignancies, including melanomas, anaplastic carcinomas, and other types of sarcomas, which typically remain negative for UEA-I (226).

#### 3.4.14 PD-L1

Programmed-death ligand 1 is a transmembrane protein and a member of the B7 family of type I transmembrane receptors. It plays a critical role in immune regulation and has emerged as a significant biomarker in cancer research. PD-L1 is constitutively expressed in various immune cell types (e.g., antigen-presenting cells, activated T cells, B cells, and monocytes) and certain epithelial cells, particularly under inflammatory conditions (227). In the tumor microenvironment, PD-L1 expression is upregulated on tumor cells and tumor-associated stromal cells as an adaptive immune mechanism to evade anti-tumor immune responses (228). The PD-1/PD-L1 pathway is a crucial inhibitory signaling mechanism that regulates T-cell responses and maintains peripheral tolerance (229, 230). By binding to its receptor, programmed death-1 (PD-1) on T cells, it inhibits T cell function, reduces proliferation, and can induce apoptosis (230). This interaction plays a vital role in limiting immunopathological responses in host tissues by downregulating inflammatory responses and restoring immune homeostasis. In the context of cancer, PD-L1 expression is frequently associated with immune evasion and poor clinical outcomes across multiple malignancies (230, 231). Additionally, PD-L1 engagement activates intracellular signaling pathways within tumor cells, including PI3K/Akt and MAPK signaling pathways, promoting cell proliferation, survival, and resistance to apoptosis (230).

In AS, studies have reported varying rates of PD-L1 positivity in AS samples, ranging from 19% to 100%, highlighting the heterogeneity of expression in this malignancy (**Table 3**). Interestingly, PD-L1 expression has been found to inversely correlate with tumor differentiation, with higher expression observed in poorly differentiated AS (232, 233). The prognostic implications of PD-L1 expression in AS are not well established, nor are their association with patient/tumor characteristics and other immune parameters. While some studies have failed to demonstrate a significant correlation between PD-L1 expression and OS (140, 232, 234), others have reported that PD-L1 overexpression may be linked to shorter survival in metastatic AS patients (144, 233, 235, 236). Notably, Honda et al. reported that PD-L1 expression was prognostic only in the context of high PD-1 positive lymphocyte infiltration (235). This discrepancy may be due to the small sample sizes in many studies, given the rarity of AS, and highlights the need for larger, multi-institutional studies to clarify the prognostic role of PD-L1.

The presence of PD-L1 expression in a substantial proportion of AS suggests that these tumors may be responsive to immune checkpoint inhibitor (ICI) therapy. In a retrospective analysis of 25 patients with AS treated with pembrolizumab monotherapy, an anti-PD-1 antibody, an objective response rate of 18%, and a disease control rate of 59% were observed, with a median PFS of 6.2 months (237). Similarly, a case series of seven patients treated with various checkpoint inhibitors reported partial responses in 71% of patients in 12 weeks (238). These findings highlight the potential efficacy of PD-1/PD-L1 inhibitors in AS, particularly in cases with high PD-L1 expression. However, the variability in response rates underscores the need for larger prospective clinical trials to systematically evaluate the therapeutic efficacy of ICIs as monotherapy or in combination with other agents. Such studies are essential for optimizing treatment strategies for this aggressive vascular malignancy and for identifying predictive biomarkers to stratify patients who are most likely to benefit from immunotherapy.

## 4 Discussion

Angiosarcoma is a highly aggressive vascular malignancy characterized by rapid proliferation, early metastasis, and limited therapeutic options. This systematic review synthesizes data reported in the literature to provide a comprehensive overview of biomarkers in AS (**Figure 2**), focusing on their diagnostic, prognostic, and therapeutic implications, as well as their role in elucidating the cell origin of AS. Our synthesis highlights a complex molecular landscape defined by recurrent genetic alterations, dysregulated signaling pathways, and distinct protein expression patterns.

### 4.1 Diagnostic challenges

Accurate diagnosis of AS remains a significant clinical challenge due to its histopathological overlap with benign and malignant vascular tumors. While immunohistochemical markers such as CD31, CD34, vWF, PDPN, ERG, and VEGFR are routinely employed, none exhibit ideal specificity and sensitivity to differentiate AS from other (vascular) tumors. CD31, though highly sensitive to endothelial differentiation, is expressed in HSCs (194, 195), various leukocyte subpopulations (194), and non-AS vascular tumors (239). Similarly, CD34’s widespread expression in fibroblastic tumors and GISTs (206, 207), limits its utility in distinguishing AS from mesenchymal mimics. Lymphatic markers like VEGFR-3 and PDPN, while useful in identifying subsets of AS, are inconsistently expressed across AS cases, with PDPN also detectable in squamous cell carcinomas and seminomas (240, 241). The historical reliance on vWF is further complicated by its low sensitivity and susceptibility to serum contamination artifacts (242). Even ETS-family transcription factors, including ERG and FLI-1, despite exceptional sensitivity, lack specificity due to their expression in prostate adenocarcinoma, Ewing sarcoma, and other malignancies (151, 152, 164). These limitations underscore the necessity of employing a multi-marker panel integrating vascular (e.g., CD31, CD34, vWF, VEGFR-2) and lymphatic (e.g., podoplanin, VEGFR-3) markers to improve diagnostic accuracy in AS.

Recent advances in molecular profiling, such as detecting MYC amplification - a near-specific marker for sAS associated with prior radiation or lymphedema, highlight the critical role of integrating genomic biomarkers with IHC. This combined approach would not only aid in differentiating AS from benign mimics like atypical vascular lesions but also clarify etiologic subtypes, emphasizing the need for standardized diagnostic workflows that bridge traditional histopathology and emerging molecular techniques.

### 4.2 Prognostic implications

Prognostic stratification in AS remains clinically challenging, though emerging evidence highlights molecular and protein-level biomarkers that correlate with tumor aggressiveness and survival outcomes. Genetic alterations such as MYC amplification and PIK3CA mutations are strongly associated with aggressive disease, particularly in sAS, where MYC-amplified tumors demonstrate reduced OS (30, 46, 47). Similarly, FLT4 amplifications may drive metastatic potential through enhanced VEGF signaling, leading to decreased survival outcomes (48). Interestingly, at the protein level, diminished or absent expression of VEGFR-2, a key mediator of angiogenesis, has been linked to an unfavorable prognosis in AS (67, 89), a finding that contrasts with other malignancies where advanced disease is typically characterized by VEGFR-2 overexpression (67, 89, 93, 94). The expression levels of nuclear proteins, such as p53, Ki-67, and c-MYC, further correlate with poor prognosis and increased metastatic potential, underscoring the interplay between genomic instability, unchecked proliferation, and clinical behavior (49, 81, 91). Immune checkpoint dysregulation, evidenced by PD-L1 expression in up to 100% of AS cases, shows conflicting prognostic significance. While higher PD-L1 levels are observed in poorly differentiated AS tumors (232, 233), their association with survival remains inconsistent, possibly due to anatomic site-specific variability or tumor microenvironment heterogeneity. Overall, the prognostic significance of AS biomarkers highlights the need for future research to explore various factors for improving overall survival.

### 4.3 Therapeutic strategies

The therapeutic implications of biomarkers in AS highlight potential targeted approaches for this aggressive malignancy. Current therapeutic strategies primarily focus on inhibiting various components of metabolic pathways implicated in AS pathogenesis. The VEGF pathway, central to AS pathogenesis, is a key target due to frequent overexpression of VEGF-A and VEGFR-1,2,3. Targeted therapies such as VEGF inhibitors (e.g., bevacizumab) (97, 98) and VEGFR inhibitors (e.g., sorafenib, pazopanib, regorafenib) (99–101) have shown modest clinical efficacy. Combination therapies, such as VEGFR inhibitor (cediranib) with MEK inhibitor (trametinib), may enhance treatment responses (114). The PI3K/Akt/mTOR and MAPK/ERK pathways, frequently activated in AS, present additional therapeutic targets. Two FDA-approved PI3K inhibitors, alpelisib, and copanlisib, are available for the treatment of various cancers and may hold promise for AS (119, 120). In a mouse model of AS, combined inhibition of PI3K/Akt/mTOR and MAPK/ERK pathway using rapamycin and trametinib led to sustained tumor regression compared to monotherapy (115). KIT inhibitors, such as imatinib, offer another potential therapeutic option for soft tissue AS (213). Additionally, the expression of PD-L1 in AS raises the possibility for immunotherapeutic approaches, though further research is needed to establish their efficacy in this context (237, 238). These biomarker-driven therapeutic strategies offer hope for improving outcomes in AS. However, the rarity of AS and its molecular heterogeneity pose challenges for conducting large-scale clinical trials. Future research should focus on validating these potential targets and exploring combination therapies that address the complex molecular landscape of AS.

### 4.4 Cell origin of angiosarcoma

The precise cellular origin of AS remains a subject of ongoing debate in the scientific community. While the endothelial origin of AS is well-established, uncertainty persists regarding whether AS originates from blood vessels, lymphatic vessels, or their respective progenitor cells. The ubiquitous presence of endothelial cells throughout the body explains why AS can arise from multiple locations. This diversity in anatomical sites of origin contributes to the complexity of determining the exact cellular lineage from which AS develops. Immunohistochemical studies have provided some insights into the potential origin of AS. The expression of both blood vascular markers (e.g, CD31, CD34, ERG, vWF, VEGFR-2) and lymphatic markers (e.g., podoplanin, VEGFR-3) in AS suggests a hybrid endothelial phenotype (83, 91, 216). This co-expression of angiogenic and lymphangiogenic markers supports the notion that AS represents a heterogeneous group of tumors with diverse endothelial origins. Additionally, CD117 expression in AS has been proposed to reflect an oncofetal phenotype, suggesting that AS cells may retain features of embryonic endothelial precursors (210). CD117 is expressed in immature cells, including HSCs and early endothelial progenitor cells (EPCs), with its expression decreasing in late EPCs and absent in mature endothelial cells (243, 244). In contrast, CD34 expression is not limited to HSCs or early EPCs; it is also detected in late EPCs and mature endothelial cells, albeit at lower levels (245). The expression of these markers, combined with the ability of AS to arise in various tissues, suggests that some cases may originate from endothelial progenitor cells (66). Understanding the cell of origin could provide valuable insights into AS pathogenesis and potentially inform the development of more targeted therapeutic strategies.

### 4.5 Limitations

The included studies face several important limitations that warrant consideration. The rarity of AS restricts the availability of large-scale studies, resulting in most included studies having small sample sizes. This limits the statistical power to detect significant associations between biomarkers and clinical outcomes. The considerable heterogeneity of AS in terms of etiology, anatomical locations, and histological subtypes further complicates the identification and validation of reliable biomarkers across different AS subgroups. Furthermore, variations in study design, patient populations, and methodologies for biomarker detection make direct comparisons between studies challenging. While numerous genetic alterations and protein expression patterns have been identified, the functional significance of many biomarkers in AS pathogenesis remains poorly understood, hampering the translation of these discoveries into diagnosis and targeted therapeutic strategies. Additionally, the rapidly evolving molecular understanding of AS, as evidenced by recent studies revealing distinct genomic profiles based on tumor primary site (23, 26, 28), makes it difficult to establish a definitive biomarker panel universally applicable across all AS subtypes. Finally, publication bias, which favors studies with positive or statistically significant findings, is a potential concern. This bias may lead to an overestimation of the prognostic or therapeutic value of certain biomarkers in the published literature.

### 4.6 Conclusion

Angiosarcoma is an aggressive and challenging malignancy, and the identification of reliable tissue biomarkers would be crucial for improving diagnosis, prognosis, and treatment strategies. Immunohistochemical markers panels including both vascular and lymphatic markers are essential for accurate diagnosis, while prognostic markers such as Ki-67, p53, PD-L1 provide insights into disease progression. Advances in molecular profiling have identified key angiogenic and oncogenic pathways, including VEGF, ANGPT-TIE, PI3K/Akt/mTOR, and MAPK/ERK, as potential therapeutic targets. However, the clinical utility of these biomarkers remains limited due to the rarity and heterogeneity of AS, as well as the inconsistency in study design and methodology. Further research should focus on conducting large-scale with standardized protocols to validate these findings. Continued efforts in biomarker discovery and targeted therapy development may improve patients’ outcomes in this aggressive malignancy.

## Data Availability

The original contributions presented in the study are included in the article/**Supplementary Material**. Further inquiries can be directed to the corresponding author.

## Appendix A:

**PubMed:** (“Biomarkers” [Mesh] OR “Biomarker” [TIAB] OR “Marker” [TIAB] OR “Parameter” [TIAB] OR “Value” [TIAB]) AND (“Hemangiosarcoma” [Mesh] OR “Angiosarcoma” [TIAB]) AND (“Humans” [Mesh] OR “Humans” [TIAB]).

**Embase:** (‘biomarker’/exp OR ‘biomarker’:ti,ab OR ‘marker’:ti,ab OR ‘parameter’:ti,ab OR ‘value’:ti,ab) AND (‘angiosarcoma’/exp OR ‘angiosarcoma’:ti,ab) AND (‘human’/exp OR ‘human’:ti,ab).
